# Calcineurin signaling pathway influences *Aspergillus niger* biofilm formation by affecting hydrophobicity and cell wall integrity

**DOI:** 10.1186/s13068-020-01692-1

**Published:** 2020-03-16

**Authors:** Li Liu, Bin Yu, Wenjun Sun, Caice Liang, Hanjie Ying, Shengmin Zhou, Huanqing Niu, Yibing Wang, Dong Liu, Yong Chen

**Affiliations:** 1 National Engineering Research Center for Biotechnology, College of Biotechnology and Pharmaceutical Engineering, Nanjing Tech University, Nanjing, China; 2 State Key Laboratory of Materials-Oriented Chemical Engineering, College of Biotechnology and Pharmaceutical Engineering, Nanjing Tech University, Nanjing, 211816 People’s Republic of China; 3 School of Chemical Engineering and Energy, Zhengzhou University, Zhengzhou, 450001 China; 4 State Key Laboratory of Bioreactor Engineering, School of Biotechnology, East China University of Science and Technology, Shanghai, 200237 China

**Keywords:** *Aspergillus niger*, Biofilm, Calcineurin signaling pathway, Cell wall integrity, Hydrophobicity

## Abstract

**Background:**

Biofilms, as a kind of fixed-cell community, can greatly improve industrial fermentation efficiency in immobilized fermentation, but the regulation process is still unclear, which restricts their application. Ca^2+^ was reported to be a key factor affecting biofilm formation. However, the effect of Ca^2+^ on biofilm structure and microbiology was yet only studied in bacteria. How Ca^2+^-mediated calcineurin signaling pathway (CSP) alters biofilm formation in bacteria and fungi has rarely been reported. On this basis, we investigated the regulation of CSP on the formation of biofilm in *Aspergillus niger*.

**Results:**

Deletion of the key genes *MidA*, *CchA*, *CrzA* or *CnaA* in the CSP lowered the Ca^2+^ concentration in the mycelium to a different extent, inhibited the formation of *A. niger* biofilm, reduced the hydrophobicity and adhesion of spores, destroyed the cell wall integrity of hyphae, and reduced the flocculation ability of hyphae. qRT-PCR results showed that the expression of spore hydrophobic protein *RodA*, galactosaminogalactan (GAG) biosynthesis genes (*uge3*, *uge5*, *agd3*, *gtb3*), and α-1,3-glucan biosynthesis genes (*ags1*, *ags3*) in the ∆*MidA*, ∆*CchA*, ∆*CrzA*, ∆*CnaA* strains were significantly down-regulated compared with those of the wild type (WT). In addition, the transcription levels of the chitin synthesis gene (*chsB*, *chsD*) and β-1,3-glucan synthesis gene (*FksA*) were consistent with the change in chitin and β-1,3-glucan contents in mutant strains.

**Conclusion:**

These results indicated that CSP affected the hydrophobicity and adhesion of spores, the integrity of mycelial cell walls and flocculation by affecting Ca^2+^ levels in mycelium, which in turn affected biofilm formation. This work provides a possible explanation for how CSP changes the formation of *A. niger* biofilm, and reveals a pathway for controlling biofilm formation in industrial immobilized fermentation.

## Background

Compared with free-cell fermentation, biofilm-based immobilized cell fermentation can not only reuse cells, reduce costs, but also increase production efficiency and shorten fermentation cycles [1, 2]. However, the biofilm formation process is extremely complex, which seriously restricts the application of biofilm-based immobilized cell fermentation. Therefore, exploring the mechanism of biofilm formation has received more and more attention. In recent years, we have conducted effective studies on the biofilm-based batch or continuous fermentation of *Saccharomyces cerevisiae*, *Penicillium citrinum*, *Corynebacterium glutamicum*, *Clostridium acetobutylicum*, and *Escherichia coli* [3–7]. However, for *A. niger*, the main strain used for industrial citric acid production, although we have developed a relatively optimal immobilization carrier, the mechanism of biofilm formation in the fermentation process has not been well studied [8].

As a highly structured microbial community, biofilms formation usually goes through three stages of attachment, maturation, and dispersion [9]. However, filamentous fungi, due to the particularity of the structure and function of their spores and hyphae, cause the formation of biofilms to be different from bacteria and yeast [10]. Namely, the formation stage of filamentous fungi in biofilms includes spore attachment, germination, hyphal elongation, colonization, production of the extracellular matrix, maturation and diffusion of biofilms [11]. Generally, attachment ability of spores is the key to whether filamentous fungi can form biofilms, which involves complex interactions between physical and biological processes [12]. Secondly, cross-linking between hyphae plays an important role in the production of extracellular matrix (ECM) in the middle stage of biofilm formation. It results in the formation of filamentous fungal biofilms that differ from bacteria and yeast.

**Fig. 1:**
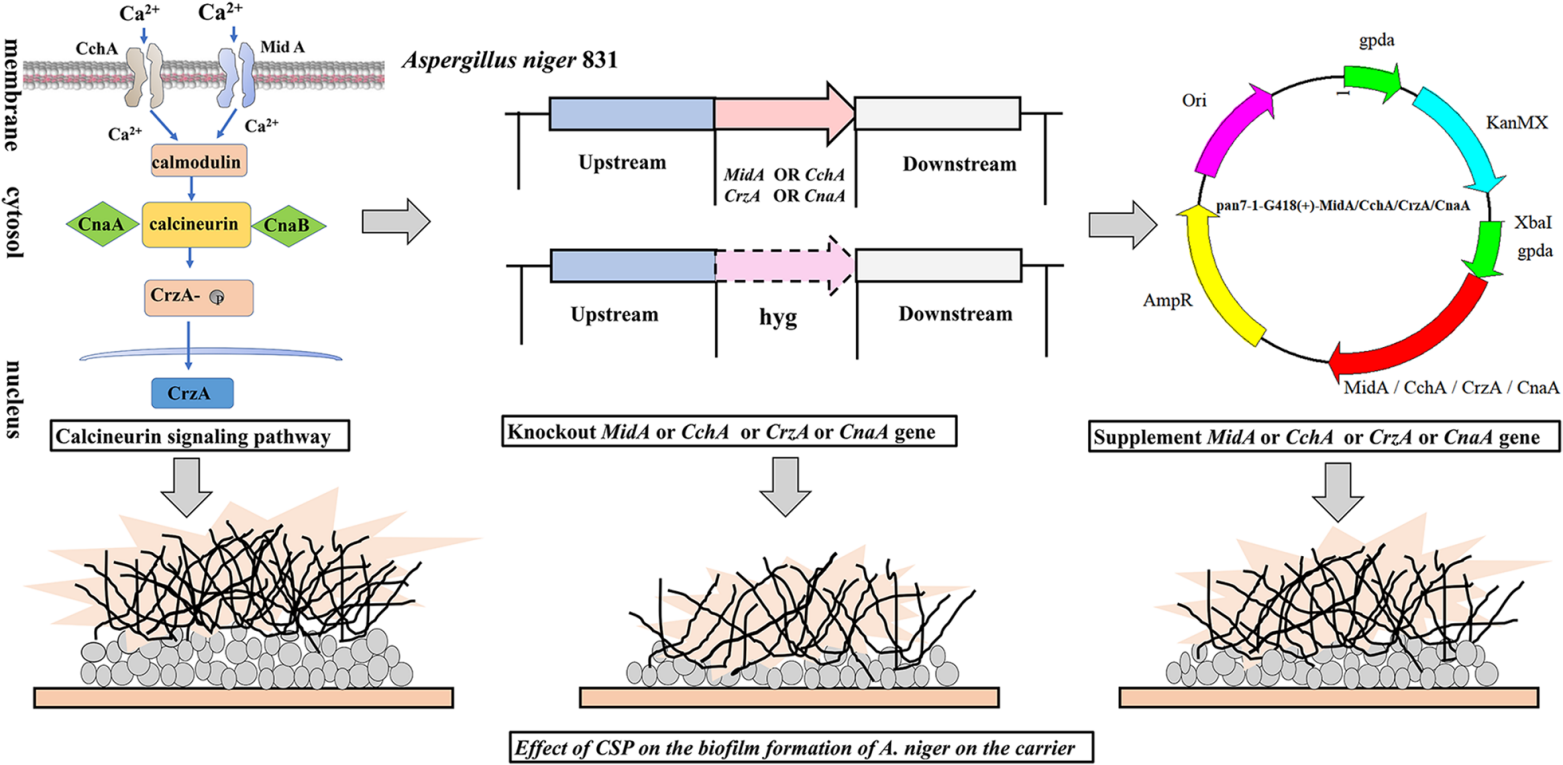
Schematic diagram of the roles of *MidA*, *CchA*, *CrzA* and *CnaA* in CSP, and their gene knockout and complementation in *A. niger*

Biofilm formation is affected by a variety of external and internal factors. Quorum-sensing molecules are the intrinsic factor of biofilm formation, while pH, temperature, nutrients, metal ions are common external factors affecting biofilm formation [13, 14]. As external factors, metal ions have recently gained attention for their role in biofilm formation. Cu^2+^, Zn^2+^, Fe^2+^, Fe^3+^ and Al^3+^ protect *Bacillus subtilis* biofilms from erosion [15]. In yeast, sub-inhibitory concentrations of Co^2+^, Zn^2+^, Cd^2+^, Hg^2+^, Pb^2+^ could cause changes in biofilm [16]. However, Ca^2+^ acts as an important second messenger in cells. At present, only a small amount of literature reports on the effect of Ca^2+^ concentration on biofilm structure and microbiology in bacteria [17, 18]. CSP is the major Ca^2+^-mediated signaling pathway. However, in filamentous fungi, the study of the Ca^2+^ channel *MidA*/*CchA*, calcineurin catalytic subunit *CnaA*, and transcription factor *CrzA* in this signaling pathway was limited to virulence, drug targets, and functional aspects. In *Aspergillus nidulans*, *MidA* and *CchA* play an important role in the regulation of mycelial polarity and conidia in a low-calcium environment, and deletion of *MidA* and *CchA* in *Aspergillus fumigatus* can lead to a decrease in virulence [19, 20]. In fungal, the loss of *CrzA* and *CnaA* results in a decrease in conidia and a decrease in tolerance to high Ca^2+^ concentration, alkaline pH, cell wall and temperature stresses [21–24]. Although this has not been reported in *A. niger*, we speculate that this regulatory pathway is also present in *A. niger*, and that CSP may be involved in the formation of *A. niger* biofilm (Fig. 1).

In this context, an accurate understanding of the regulation of biofilm formation by the CSP is critical in the immobilization fermentation processes. In this study, *A. niger* biofilm formation was first found to be apparently reduced upon knocking out the genes in CSP. Finally, it turned out that CSP controlled the hydrophobicity of spores, the integrity of cell walls, and the flocculation of hyphae predominantly by controlling the Ca^2+^ contents in *A. niger* mycelium, thereby controlled the formation of biofilm.

## Results

### Ca^2+^ levels in mycelium affect biofilm formation of *MidA*, *CchA*, *CrzA* and *CnaA* strains

Metal ions are considered to be important factors influencing the formation of microbial biofilms [15, 16]. To investigate whether Ca^2+^ levels in mycelium mediated changes in biofilm formation, we inactivated *MidA*, *CchA*, *CrzA* and *CnaA* in CSP, respectively, and measured total Ca^2+^ levels in mycelium of WT, mutant, and complemented strains. As shown in Table 1, the deletion of *MidA*, *CchA*, *CrzA* or *CnaA* significantly reduced the total Ca^2+^ contents in mycelium, while complementation of these genes effectively recovered the total Ca^2+^ contents in mycelium. This result indicated that the CSP was indeed effective in regulation Ca^2+^ in mycelium and disruption of CSP would decrease the Ca^2+^ contents in *A. niger* mycelium.

**Table 1:** Calcium content in mycelium of different strains

	Strain	Total Ca^2+^ contents
Calcium (mg/kg dry cell weight)	Wild type	125
	∆*MidA*	65.8
	∆*CchA*	45.7
	∆*CrzA*	70.0
	∆*CnaA*	74.1
	*MidAC*	109
	*CchAC*	107
	*CrzAC*	91.7
	*CnaAC*	105

**Fig. 2:**
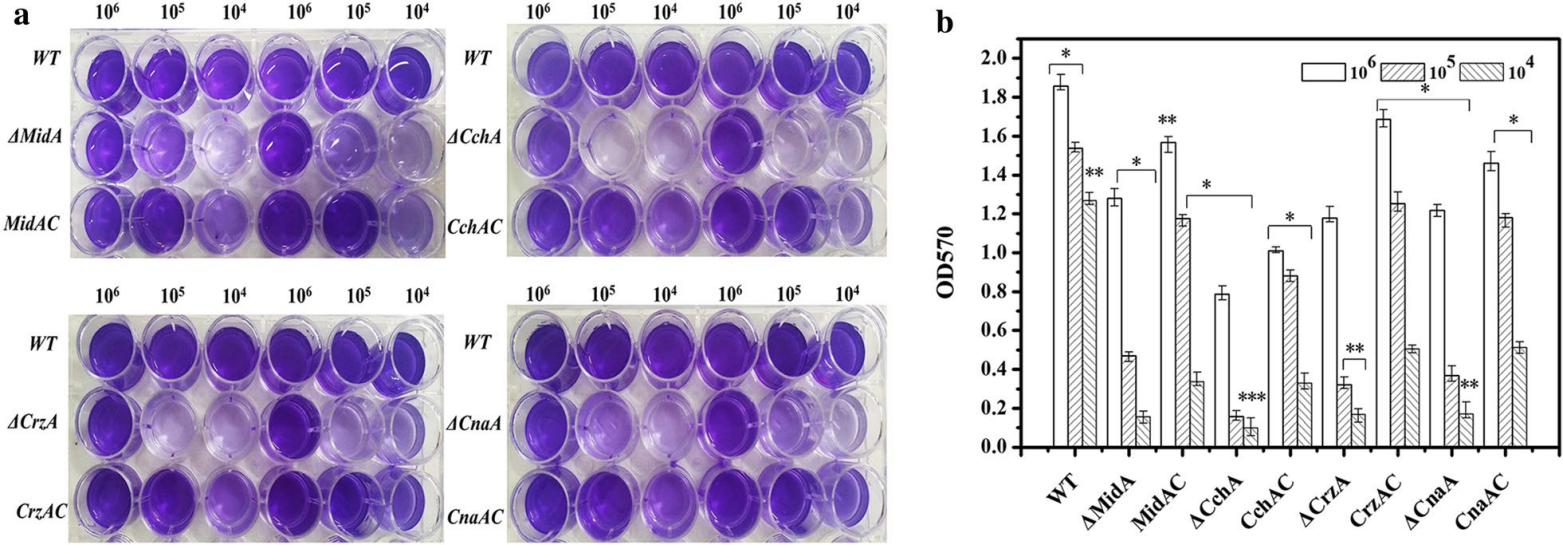
Biofilm formation. **a** Wild type, ∆*MidA*, ∆*CchA*, ∆*CrzA*, ∆*CnaA*, *MidAC*, *CchAC*, *CrzAC* and *CnaAC* strains were incubated in 24-well plates for 48 h and their adhesion ability was examined. The free cells were removed, washed 3 times with PBS (1 mL), and stained with 0.1% crystal violet. Wells were repeatedly washed with water, dissolved with acetic acid and photographed. **b** Adhesion was expressed as OD_570_ and was measured by solubilizing crystal violet in acetic acid. The values are the means and standard deviations of three independent experiments. ***p < 0.001, **p < 0.01, *p < 0.05 by Student’s *t* test

**Fig. 3:**
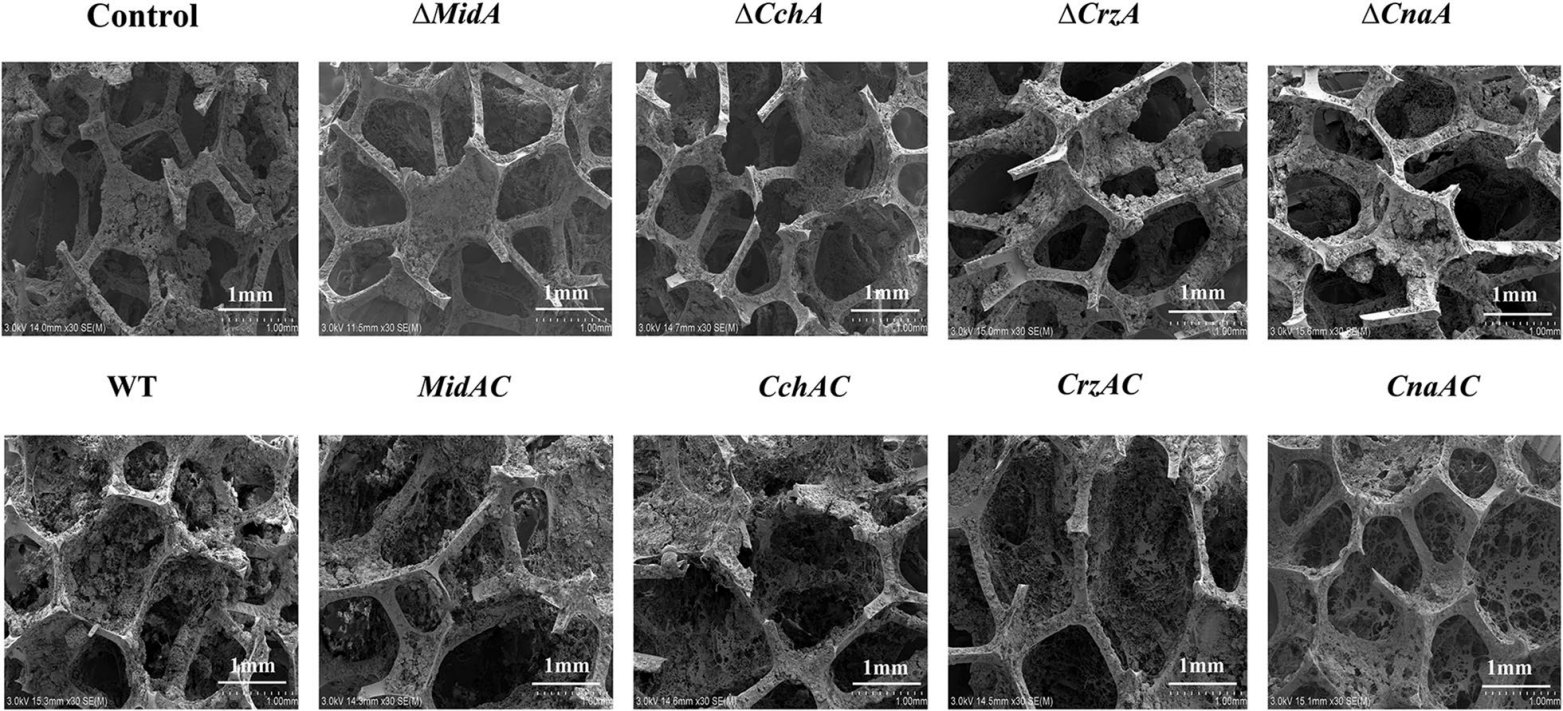
Biofilm formation. SEM images of biofilms were formed on carbon brazed carriers after fermentation of WT, ∆*MidA*, ∆*CchA*, ∆*CrzA*, ∆*CnaA*, *MidAC*, *CchAC*, *CrzAC* and *CnaAC* strains in synthetic medium for 48 h. The magnification was 30 times

To evaluate the ability of each strain to form biofilms on a solid surface, biofilms grown in 24-well plates were quantified using CV assay (Fig. 2a, b). Biofilm formation was reduced in all gene deletion mutants compared to WT. Among these mutant strains, ∆*CchA* formed the least biofilm. When the spore amount was 10^5^, the biofilm content of ∆*MidA*, ∆*CchA*, ∆*CrzA*, ∆*CnaA* were 30.55%, 10.25%, 20.89% or 23.98% of WT, respectively. The biofilms of gene complemented strains *MidAC*, *CchAC*, *CrzAC* or *CnaAC* were recovered to 76.55%, 57.29%, 81.56% or 76.91% of WT, respectively. To further confirm this finding, SEM was used to observe the biofilm on the carrier during fermentation (Fig. 3) and the results were consistent with those of CV assay. The amount of biofilm on the carrier of the four mutant strains was significantly reduced relative to the WT and the complemented strains.

Collectively, CSP disruption decreased Ca^2+^ levels in mycelium and reduced biofilm formation. This indicated that CSP controlled the formation of *A. niger* biofilm by controlling changes in Ca^2+^ concentration in mycelium.

### *A. niger* ∆*MidA*, ∆*CchA*, ∆*CrzA*, ∆*CnaA* strains reduced the hydrophobicity and adsorption properties of spores

**Fig. 4:**
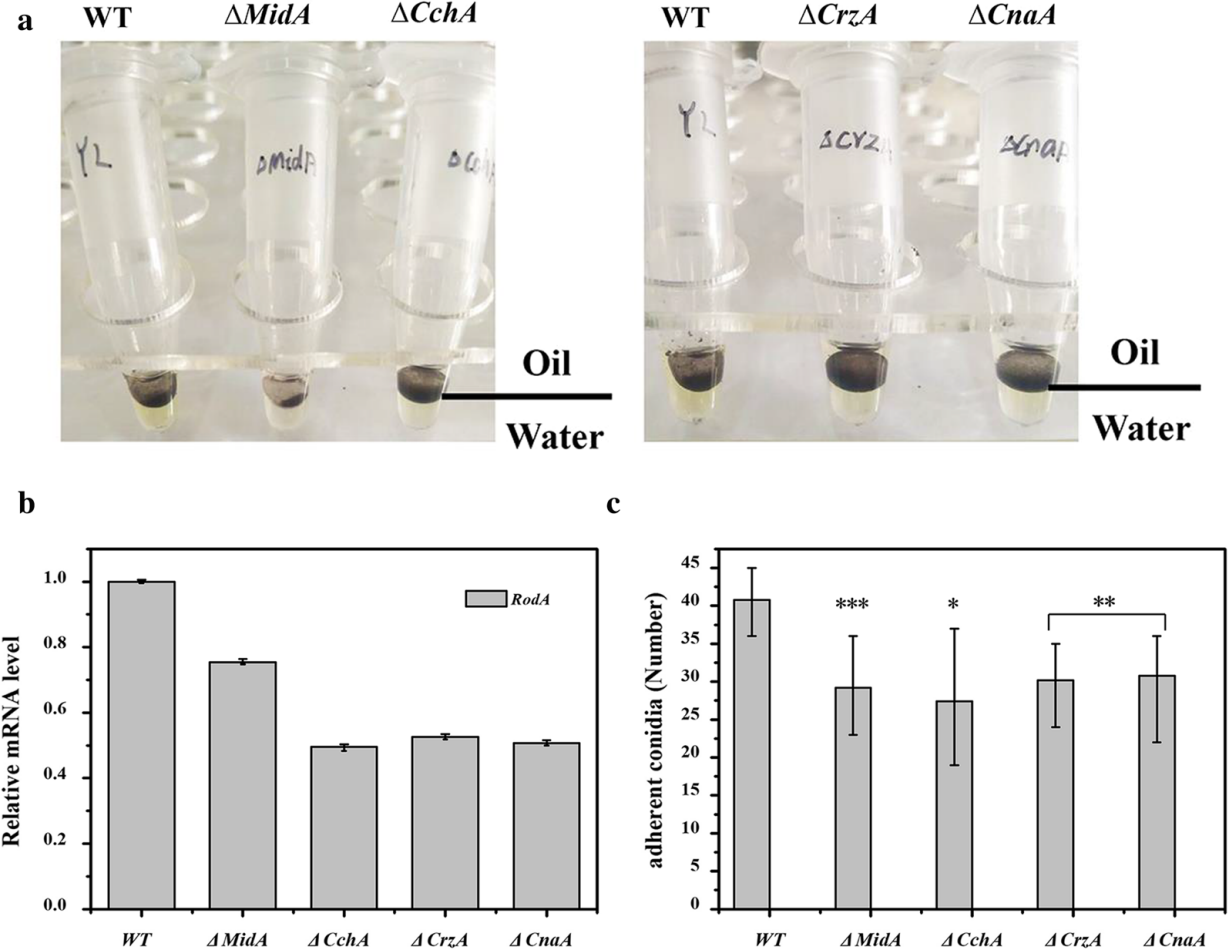
Aspergillus niger *MidA*, *CchA*, *CrzA*, *CnaA* strains reduced adhesion properties. **a** Shown are WT and ∆*MidA*, ∆*CchA*, ∆*CrzA*, ∆*CnaA* conidia in a 1:1 water–oil (glyceryl tributyrate) interface. **b** qRT-PCR results. Relative expression of *RodA* genes in ∆*MidA*, ∆*CchA*, ∆*CrzA* and ∆*CnaA* compared with WT, respectively. **c** Adhesion properties of *A. niger* wild type and mutant strains on glass slides. Adhesion number of conidia on coverslips. The values are the means and standard deviations of three independent experiments. ***p < 0.001, **p < 0.01, *p < 0.05 by Student’s *t*-test

Spores surface hydrophobicity and adhesion are preconditions for the formation of biofilms [25, 26]. Therefore, the mechanism of CSP regulating the formation of *A. niger* biofilm was further investigated by evaluating the hydrophobicity and adhesion of the mutant strains. In the hydrophobicity experiment, spores of WT and mutant strain were separately added to the hydrophobic layer of glyceryl tributyrate and left to stand (Fig. 4a). The color of the hydrophobic layer of spores with strong hydrophobicity will become dark, while the color of the hydrophobic layer of spores with weak hydrophobicity will become light. Compared to WT, the color in the hydrophobic layer of the *∆MidA*, *∆CchA*, *∆CrzA* and *∆CnaA* mutants was significantly lighter. *RodA* gene expresses a hydrophobic protein and is the main source of hydrophobic force on the surface of spores [27]. So, the expression level of *RodA* in the mutant strain was quantitatively detected by qRT-PCR (Fig. 4b). Compared with WT, the expression levels of hydrophobin *RodA* in ∆*MidA*, *∆CchA*, *∆CrzA* or *∆CnaA* mutants were significantly down-regulated.

To further explore the effect of the mutant strains in the initial stage of biofilm formation, after the initial adhesion stage of 6 h, the conidia adsorbed on the coverslip surface were directly quantified by microscopic counting (Fig. 4c). The results showed that there were significant differences in the adhesion ability between the four mutant strains and the WT strain. The number of WT conidia adsorbed on coverslips was significantly higher than that of the four mutant strains (Additional file 1: Fig S1). Collectively, these results demonstrated that the CSP disruption in the mutant strains reduced the hydrophobicity and adhesion of the conidia, thereby affecting the initial formation of biofilm.

### *A. niger* ∆*MidA*, ∆*CchA*, ∆*CrzA*, ∆*CnaA* strains have impaired cell wall integrity

**Fig. 5:**
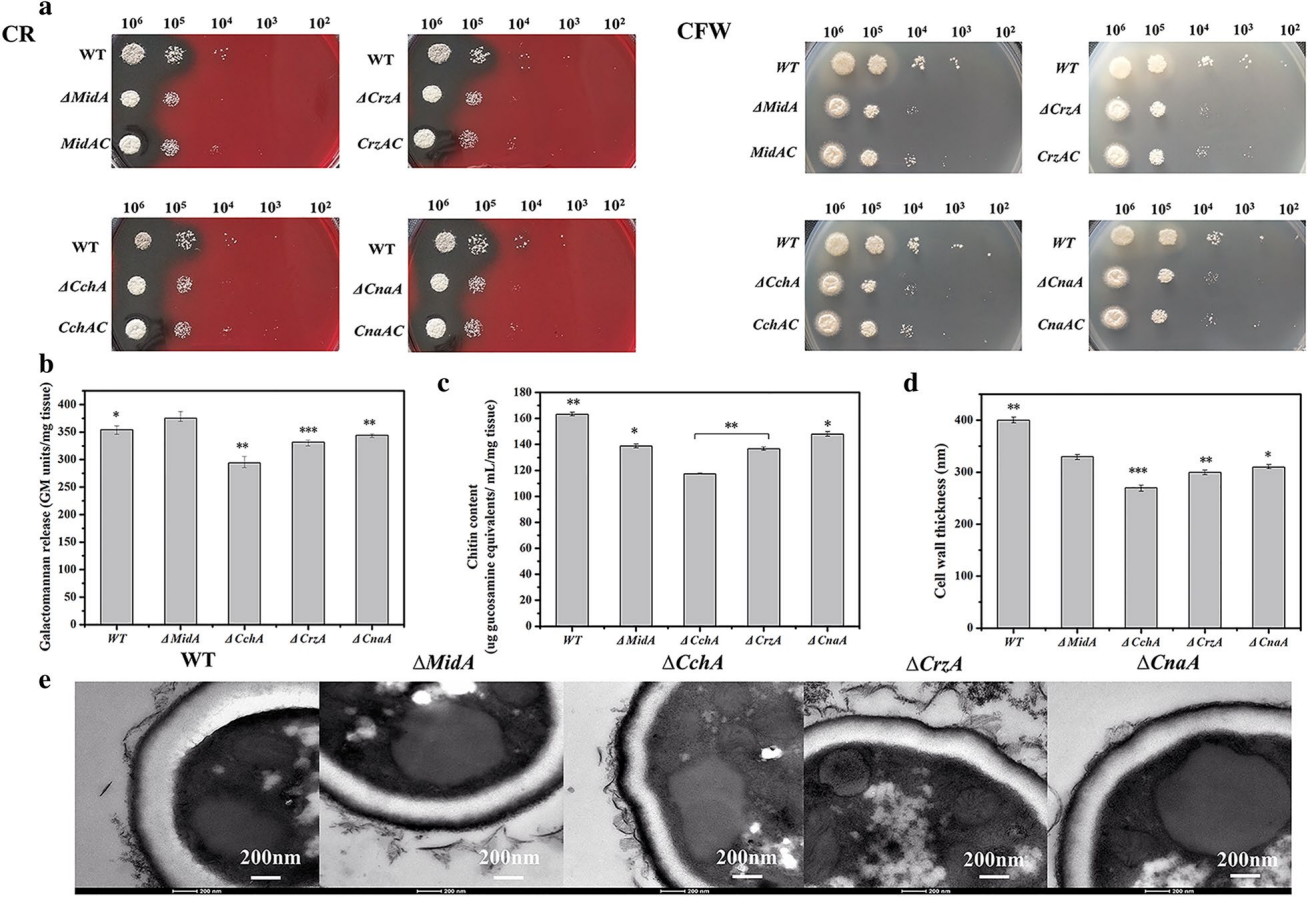
*A. niger* ∆*MidA*, ∆*CchA,* ∆*CrzA* and ∆*CnaA* strains disrupted cell wall integrity. **a** Comparison of resistance of *MidA*, *CchA*, *CrzA*, *CnaA*, *MidAC*, *CchAC*, *CrzAC*, *CnaAC* and wild type strains to cell wall disruptors. **b**, **c** Comparison of chitin, β-1,3-glucan contents between wild type and mutant. **d** Cell wall thickness of mutant and WT. **e** TEM images of *A. niger* WT, ∆*MidA*, ∆*CchA*, ∆*CrzA* and ∆*CnaA* strains. TEM analysis (bars, 200 nm). The values are the means and standard deviations of three independent experiments. ***p < 0.001, **p < 0.01, *p < 0.05 by Student’s *t* test

Crosslinking between hyphae plays an important role in the production of ECM in the middle stage of biofilm formation, and crosslinking between hyphae is usually associated with changes in carbohydrate components in the cell wall. Fungal cell walls are mainly composed of covalently connected polysaccharide backbone (glucans, chitin) interlaced and coated with glycoproteins [28]. Congo red (CR: 800 μg/mL) and Calcofluor white (CFW: 400 μg/mL) have been widely used as indicators for showing cell wall defects. Here, we investigated the susceptibility of ∆*MidA*, ∆*CchA*, ∆*CrzA*, ∆*CnaA* mutants and complemented strains *MidAC*, *CchAC*, *CrzAC*, *CnaAC* to these two cell wall disrupters (Fig. 5a). It was found that *MidA*, *CchA*, *CrzA* and *CnaA* mutant strains were more sensitive to the cell wall disrupter CR and CFW than WT and complemented strains, indicating that the cell wall composition of the mutant strains might have changed.

Since chitin and β-1,3-glucan are the major structural components of the fungal cell wall, both components of the mutant strain and the WT were quantitatively analyzed. The chitin and β-1,3-glucan in ∆*MidA*, ∆*CchA*, ∆*CrzA* and ∆*CnaA* mutants were 85.09% and 105.84%, 71.95% and 82.90%, 83,71% and 93.56%, 90.58 and 97.15% of WT, respectively (Fig. 5b, c). TEM analysis (Fig. 5d, e) showed that the cell wall thickness of ∆*MidA*, ∆*CchA*, ∆*CrzA*, and ∆*CnaA* was 82.5%, 67.5%, 75.0%, and 77.5% of WT, respectively. These results again indicated that *MidA*, *CchA*, *CrzA*, and *CnaA* played a role in cell wall composition.

**Fig. 6:**
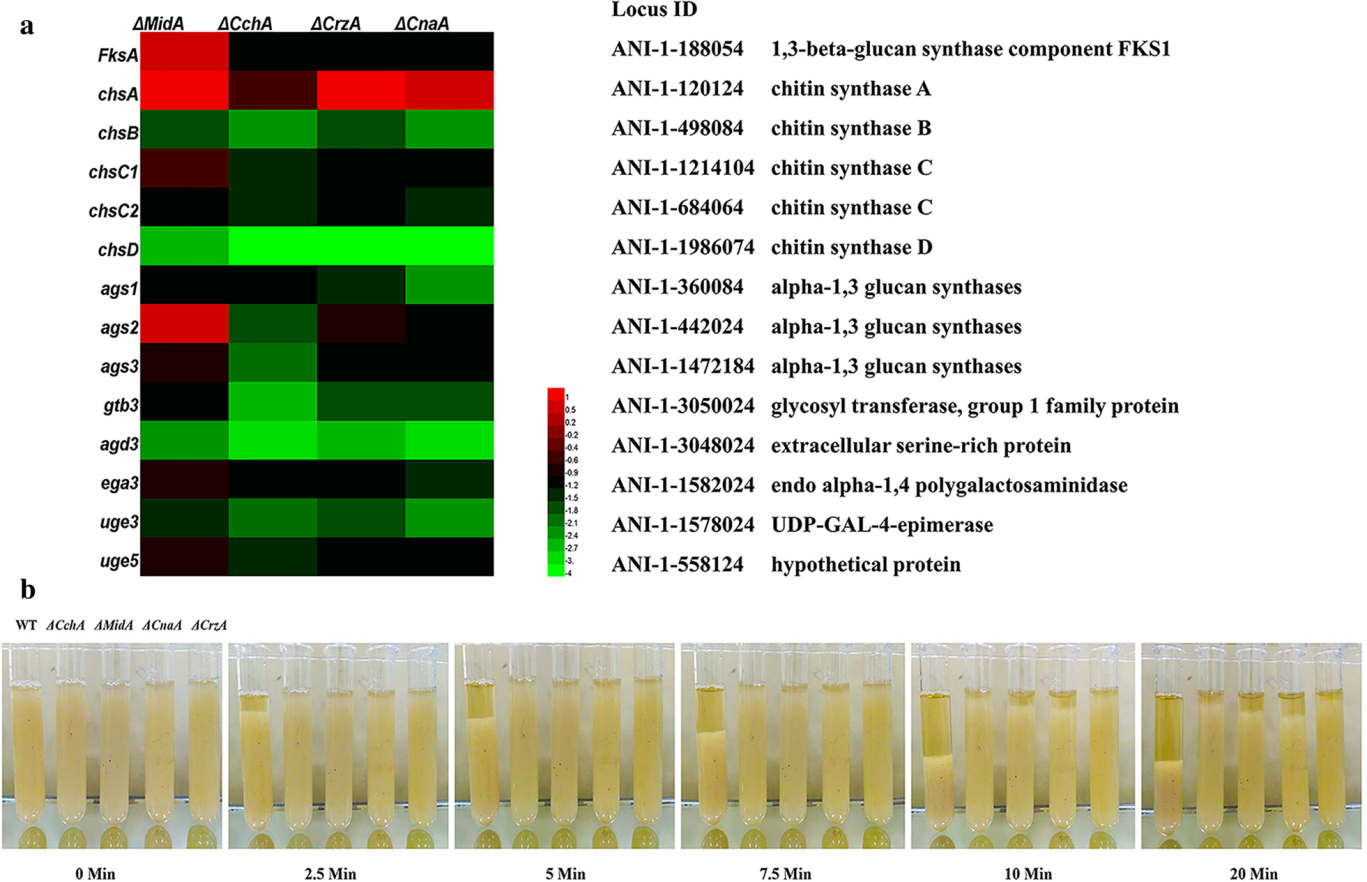
qRT-PCR result and flocculation ability. **a** Differences in expression levels of chitin, β-1, 3-glucan, α-1,3-glucan and GAG-related genes in mycelia cell wall in WT, ∆*MidA*, ∆*CchA*, ∆*CrzA* and ∆*CnaA* strains. **b** WT, ∆*MidA*, ∆*CchA*, ∆*CrzA* and ∆*CnaA* strains were photographed every 2.5 min

In addition to chitin and β-1,3-glucan, the components of the fungal cell wall are also mainly composed of α-1,3-glucan, GAG, and galactomannan. α-1,3-Glucan has been shown to play an important adhesion role in the interaction of mycelium, which is synthesized by α-1,3-glucan synthetase encoded by *ags1, ags2* and *ags3* genes [29]. GAG is characterized by its dual functions of adhesion and virulence. It has been proved to play a key role in the formation of biofilms [30]. The key genes in its synthetic pathway are *uge3*, *uge5*, *agd3, ega3, gtb3, sph3,* while *uge3* and *uge5* are also necessary for galactomannan synthesis. In addition, *chsA, chsB, chsC,* and *chsD* genes encode chitin synthetase and *FksA* encodes β-1,3-glucan synthase, which plays a crucial role in maintaining the cell wall integrity. To determine whether the expression of the genes associated with these polysaccharide components has changed in the mutant strains, the mRNA levels of these genes were analyzed (Fig. 6a). The results showed that the mRNA levels of GAG and α-1,3-glucan related genes *uge3*, *uge5*, *agd3*, *ega3* and *gtb3* or *ags2* and *ags3* were significantly reduced in the four mutant strains. In addition, the expression levels of two chitin synthesis encoding genes, *ChsC* and *ChsD*, were also down-regulated, indicating that the deletion of *MidA*, *CchA*, *CrzA*, *CnaA* had inhibitory effects on these genes. Interestingly, the expression levels of the β-1,3-glucan synthetase encoded gene *FksA* was up-regulated in the *MidA* mutant, while down-regulated in the *CchA*, *CrzA* and *CnaA* mutants, which was consistent with the test results of β-1,3-glucan content in the mutant.

To explore whether *MidA*, *CchA*, *CrzA*, and *CnaA* genes affect the polysaccharide components in biofilms, the expression levels of polysaccharide components related genes in the biofilm were also measured via qRT-PCR (Additional file 2: Fig S2). The *uge3*, *agd3*, *gtb3*, *ags1* and *chsB* were down-regulated in ∆*MidA*, ∆*CchA*, ∆*CrzA*, and ∆*CnaA* mutants to vary degrees. This indicated that the polysaccharide component in the biofilm had also changed. It also showed that CSP could affect biofilm formation by affecting cell flocculation. So, flocculation ability between the mutant strains and WT strain was compared (Fig. 6b). Results showed that the ∆*MidA*, ∆*CchA*, ∆*CrzA*, and ∆*CnaA* mutants lost most of its flocculation ability. It could conclude that in ∆*MidA*, ∆*CchA*, ∆*CrzA* and ∆*CnaA*, the loss of flocculation ability and cell wall integrity impaired cell adhesion and affected the formation of biofilm.

### Effects of *MidA*, *CchA*, *CrzA*, *CnaA* strains on immobilized and free cell fed-batch fermentation

**Fig. 7:**
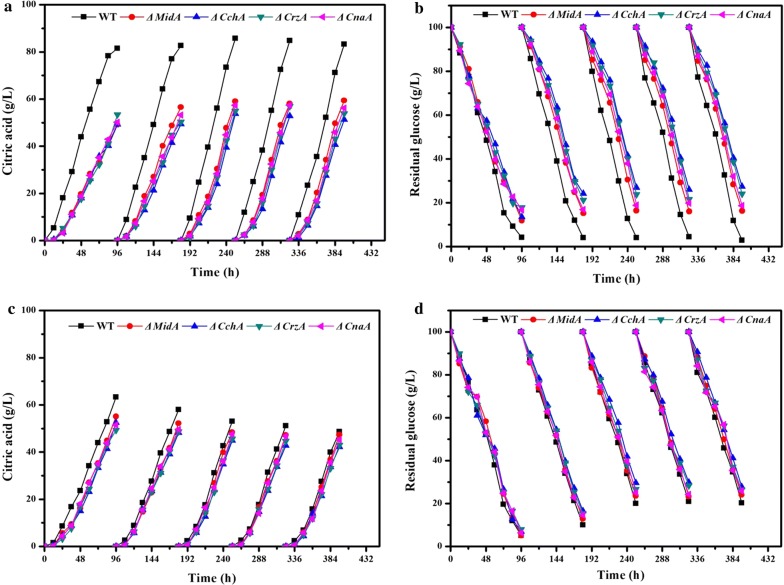
Changes in citric acid and glucose concentrations during fermentation. **a**, **b** Biofilm fermentation. **c**, **d** free-cell fermentation

Formation of biofilms is key to immobilize fermentation. To explore the effects of biofilm reduction in ∆*MidA*, ∆*CchA*, ∆*CrzA*, ∆*CnaA* on the production of citric acid, the citric acid yield and glucose consumption of the mutants in free-cell and biofilm-based fed-batch fermentation were compared (Fig. 7). In free-cell fermentation, the ∆*MidA*, ∆*CchA*, ∆*CrzA*, ∆*CnaA* strains and the WT strain showed almost similar performances. However, in biofilm fermentation, ∆*MidA*, ∆*CchA*, ∆*CrzA*, ∆*CnaA* strains consumed glucose and produced citric acid slightly slower than wild type strain in the first batch. In the next four batches of biofilm fermentation, both the yield of citric acid and sugar consumption rate of ∆*MidA*, ∆*CchA*, ∆CrzA, and ∆CnaA strains were lower than those of wild type strain. By contrast, in the free-cell fermentation, *∆MidA*, ∆*CchA*, ∆*CrzA*, ∆*CnaA* and WT strains consumed glucose and produced citric acid almost the same. This indicated that the decrease in citric acid yield in *A. niger* in immobilized fermentation resulted from biofilm reduction that was caused by the deletion of *MidA*, *CchA*, *CrzA*, or *CnaA* in CSP.

## Discussion

Due to the fact that biofilms play an important role in environmental, medical and industrial fields, understanding of biofilm formation mechanisms has become a hot spot in biofilm research. Ca^2+^ is a key metal element affecting biofilm formation. At present, a few literatures have reported the effect of Ca^2+^ on the structure and morphology of bacterial biofilms, and the Ca^2+^-mediated CSP has also been extensively studied in terms of growth, stress tolerance and virulence of fungal pathogens [17, 31, 32]. In our studies, the CSP was found to affect the formation of *A. niger* biofilms by regulating Ca^2+^ levels in mycelium. Furthermore, biofilm formation was critical for the immobilized fermentation of *A. niger*. However, there is currently no research on how CSP affects the regulation of biofilm formation.

In filamentous fungi, the CSP is an important signaling pathway regulating Ca^2+^ balance in mycelium. On this basis, we knocked out and complemented the Ca^2+^ channel MidA and *CchA*, calcineurin catalytic subunit *CnaA*, and transcription factor *CrzA* in the CSP. The deletion of *MidA*, *CchA*, *CrzA* or *CnaA* in the CSP was found to reduce Ca^2+^ contents in mycelium and reduced biofilm formation. In addition, the complementation of these genes effectively restored Ca^2+^ contents in mycelium and biofilm formation, indicating that CSP might control biofilm formation by controlling changes in Ca^2+^ contents in mycelium.

The adhesion of spores to surrounding substances, the cross-linking and attachment between hyphae, and the production of biofilms are the three basic elements of the formation of filamentous fungal biofilms [11, 12]. In fungi, the ability of conidia to adsorb on carriers is critical to immobilization (biofilm formation) [4, 8]. This adsorption mainly depends on the hydrophobic and electrostatic forces of the spores. Among them, it has been proved in *A. fumigatus* that the loss of the hydrophobic molecule *RodA* gene on the outer wall of the conidia is related to the adhesion of spores [27]. The hydrophobic ability of the conidia of Δ*MidA*, Δ*CchA*, Δ*CnaA*, and Δ*CrzA* strains was significantly lower than that of the WT, and the expression levels of Δ*MidA*, Δ*CchA*, Δ*CnaA* and Δ*CrzA* mutants hydrophobin *RodA* was consistent with the phenotype. It was indicated that *MidA*, *CchA*, *CnaA* and *CrzA* could affect the initial formation of biofilm by affecting the expression of hydrophobin *RodA*. At the same time, the adhesion ability of the conidia of mutants to the slide was significantly weaker than that of the WT. This fully proved that the more hydrophobic the conidia were, the stronger their adhesion was. These results also indicated that altered adhesion properties of conidia may be due to differences in their surface carbohydrates. Given the phenotypic similarity of the four knockout strains, it was shown that *A. niger*, like other fungi, also has this conserved signaling pathway [33]. That is, the activity of *CrzA* is dependent on the presence of calcineurin in the cell, and the presence of calcineurin depends on the presence of calcium channels *MidA* and *CchA*.

The degree of hyphae cross-linking and adhesion of hyphae is related to extracellular polysaccharides, and extracellular polysaccharides play an important role in the formation of filamentous fungal biofilms. We hypothesized that the *A. niger* cell wall and *A. fumigatus* cell wall components were similar, mainly composed of polysaccharides, such as β-1,3-glucan, chitin, GAG, α-1,3 glucan, galactomannan, maintaining cell stability and adhesive properties [34, 35]. The Δ*CchA*, Δ*CnaA*, Δ*MidA* and Δ*CrzA* mutants were more sensitive to cell wall disrupters (CR, CFW,) than WT. In addition, the mutant strains showed changes in the cell surface β-1,3-glucan and chitin content compared to the WT. Content of β-1,3-glucan content was increased in the Δ*MidA* mutant, and the chitin content was decreased, while the contents of β-1,3-glucan and chitin were reduced in the Δ*CchA*, Δ*CnaA* and Δ*CrzA* mutants. This phenotype was consistent with the transcription of relating genes. CR is a dye that prevents the formation of correct glucan. The Δ*MidA* strain was sensitive to CR, probably because CR can both block β-1,3-glucan and prevent other components from proper polymerization and accumulating on the cell wall. This fully demonstrated that absence of *MidA*, *CchA*, *CnaA* or *CrzA* in *A. niger* can affect cell wall integrity. However, the quantitative analysis of other components in the cell wall of the mutant strain need to be further studied.

The α-1,3 glucan and GAG act as adhesive substances and have been shown to be involved in the formation of biofilms [29, 30]. The key genes *ags2* and *ags3* of α-1,3 glucan synthesis was down-regulated in the Δ*MidA*, Δ*CchA*, Δ*CnaA* and Δ*CrzA* strains. *Uge3* is a key enzyme in biofilm formation, in which the deletion of *MedA* or *StuA* result to defect in biofilm, which was associated with decreased expression of *uge3* [36]. The *uge3*, *uge5*, *ega3*, *agd3*, *gtb3* gene expression levels associated with GAG were significantly reduced in the Δ*MidA*, Δ*CchA*, Δ*CnaA* and Δ*CrzA* strains. Flocculation ability depends on cell–cell adhesion [37]. Mycelial flocculation ability of the Δ*MidA*, Δ*CchA*, Δ*CnaA* and Δ*CrzA* strains was almost completely lost relative to the WT. This provided evidence for changes in mycelium adhesion and aggregation. It was suggested that the possible mechanism by which CSP affected biofilms was the interaction between cells. Importantly, expression levels of polysaccharide-related genes in biofilms were also changed. It was obvious that the change of the polysaccharide component in the mycelium caused the change in the polysaccharide component in biofilm. These results fully demonstrated that the CSP affected biofilm formation by affecting the composition of mycelial cell carbohydrates in the middle stage of biofilm formation.

## Conclusion

This study focused on the effects of CSP on the formation of *A. niger* biofilms. Results showed that the disruption of CSP could effectively decrease the content of Ca^2+^ in mycelium, it decreased biofilm formation as well. It was further revealed that *MidA*, *CchA*, *CrzA*, and *CnaA* affected the surface hydrophobicity and adhesion of *A. niger* spores in the early stage of biofilm formation, and also affected the cell wall polysaccharide composition and flocculation of hyphae, which in turn affected the ability to form biofilms. The regulatory mechanisms identified in this study can be used to regulate the formation of biofilms in immobilized fermentation.

## Materials and methods

### Strains and media

*Aspergillus niger* 831 [8] parental strain used in this study was a newly established strain which was mutagenized by the ATCC 12846 strain and maintained on conventional potato dextrose agar (PDA) plates. Its genomic sequence is not clear at present, but the base sequences of genes MidA, CchA, CrzA and CnaA were found to be the same to those from *A. niger* CBS 513.88. Therefore, in practical operation, the genome sequence of *A. niger* CBS 513.88 was taken as a reference for molecular operation. Mutant strains (Δ*MidA*, Δ*CchA*, Δ*CnaA* and Δ*CrzA*) and complemented strains (*MidAC*, *CchAC*, *CnaAC* and *CrzAC*) were cultured in solid PDA medium supplemented with 35 μg/mL hygromycin B (hyg) (31282; Sangon Biotech, China) and 50 μg/mL G418 salt (345180; Merck, Japan), respectively, incubated at 35 °C for 4 days. The fermentation medium was synthetic medium composed as follows: glucose (100 g/L), (NH_4_)_2_SO_4_ (2 g/L), NaNO_3_ (1 g/L), KH_2_PO_4_ (0.5 g/L), and MgSO_4_·7H_2_O (0.3 g/L), dissolved in 1 L of wheat bran extract. The preparation method of wheat bran extract was carried out according to a method as described previously [8]. Required amounts of wheat bran were immersed in water to obtain a suspension of 15 g/L wheat bran, which was used for subsequent hydrolysis by autoclaving at 121 °C for 60 min. The mixture was then centrifuged at 10,000*g* for 10 min to obtain wheat bran extract. For biofilm culture, the synthetic medium was used.

### Construction of Δ*MidA*, Δ*CchA*, Δ*CnaA*, Δ*CrzA MidAC*, *CchAC*, *CnaAC* and *CrzAC* strains of *A. niger*

For generating the target genes mutant strain, the fragments containing the selectable marker sandwiched by their respective upstream and downstream segments were created via overlap PCR technology. The hyg selectable marker was first amplified from plasmid PAN7-1 [38] by corresponding primers; the upstream and downstream fragments of target genes were amplified from *A. niger* genomic DNA using the corresponding primer. Then, the above three fragments were merged into gene knockout fragment by corresponding primers (Additional file 3: Table S1). Gene knockout fragments were transformed into *A. niger* by PEG-mediated protoplast transformation. Finally, transformants were selected in PDA medium containing 75 ug/mL hyg and 1 M sucrose, and the correctness of the transformant was verified by colony PCR and qRT-PCR analysis (Additional file 4: Fig S3).

To ensure that the obtained mutant phenotype can be attributed to the desired deletion, *MidA* (ANI-1-286154), *CchA* (ANI-1-1832074), *CrzA* (ANI-1-1446164) and *CnaA* (ANI-1-1832074) deletions were supplemented by the integration of *MidA*, *CchA*, *CrzA* and *CnaA* genes, respectively, to produce complementary strains *MidAC*, *CchAC*, *CrzAC* and *CnaAC*. Briefly, a DNA fragment containing gpda promoter and target fragment was cloned and inserted into PAN-7-1-G418 (stored in our lab) plasmid to generate new plasmids PAN-7-1-G418-*MidAC*, PAN-7-1-G418-*CchAC*, PAN-7-1-G418-*CrzAC*, and PAN-7-1-G418-*CnaAC*, respectively. Finally, the plasmid was transformed into four mutant strains by PEG-mediated protoplast transformation, respectively. Transformants were selected in PDA medium containing 100 ug/mL G418 salt and 1 M sucrose, and the correctness of the transformant was verified by colony PCR and qRT-PCR analysis (Additional file 1: Fig S1). All the PCR primers used above were listed in (Additional file 5: Table S2).

### Determination of calcium content in mycelium

The strains were cultured in the liquid fermentation synthetic medium for 24 h, and collected by centrifugation. The collected mycelium was washed three times with 1 uM EDTA, then washed three times with distilled water, frozen with liquid nitrogen and dried under vacuum. 0.1–1 g of sample was weighed into tetrafluoroethylene digestion tank, and dissolved adding 5 mL concentrated nitric acid at 120 °C for 20 min, followed by cooling down to room temperature. The acid-digested samples were transferred to 50-mL flasks and diluted with ultrapure water to the mark. Calcium calibration standard 2A (Agilent Technologies) diluent was prepared using the same concentration of nitric acid as the final cell extract. Ca^2+^ in samples was quantified by ICP-MS (Agilent 7500a; Agilent Technologies, USA).

### Biofilm formation on plastics

In order to determine the ability of *A. niger* strains to form biofilms, the *A. niger* crystal violet (CV) assay was performed as previously described with minor modifications [3]. 10^6^, 10^5^ and 10^4^ conidia were inoculated into 1 mL liquid synthetic medium in a 24-well microtiter plate (Corning, NY) where they were incubated for 48 h at 35 °C. Subsequently, the plate was washed 3 times with PBS to remove free cells, after which the biofilms were stained with 1 mL of 0.1% crystal violet solution for 10 min at room temperature, followed by repeated washing of the wells with PBS and air dried. Then, 1 mL of 100% acetic acid was added to each well and slightly shaken at room temperature for 30 min to elute the crystal violet. Finally, the absorbance was read at 470 nm using a microplate reader (SpectraMax Paradigm).

### Scanning electron microscopy (SEM)

Biofilms growing on the elastomeric carbon black polyurethane [8] were obtained after 48 h immobilized fermentation in the fermentation medium. Samples were washed 3 times with PBS buffer, and stored at − 80 °C. Biofilm cells were dried using a FreeZone^®^ 4.5 L Freeze Dry System (Labconco, Kansas City, MO, USA), sputtered coated with gold and viewed by scanning electron microscopy (SEM 4800, Hitachi, Japan).

### Hydrophobicity assay and flocculation assay

Frist, added 50 μL of glyceryl tributyrate in a 1.5 mL centrifuge tube, then, 50 μL of a suspension containing 10^8^ spores was carefully added above the hydrophobic layer of glyceryl tributyrate. Finally, the hydrophobicity of the spores was observed after 24 h at room temperature.

All strains were incubated with the same amounts of spores in the synthetic medium at 35 °C for 24 h. 10 mL of each strain was placed in a separate tube, and the subsequent experimental methods were as described previously [37]. The tubes were then vibrated thoroughly to suspend all contained cells and subsequently left to stand until all cells underwent sedimentation. Images were recorded every 2.5 min and flocculation ability was measured as the time required for sedimentation to complete.

### Transmission electron microscopy (TEM)

After centrifugation of the prepared spore suspension, the supernatant was removed, and the spores were washed three times with pre-cooled 2.5% glutaraldehyde fixative, and then fixed in pre-cooled 2.5% glutaraldehyde for overnight at 4 °C. Spores were fixed in 2.5% glutaraldehyde, washed three to six times with 0.1 M phosphate, post-fixed in 1% osmium tetroxide for 2 h in 4 °C, washed three times in 0.1 M phosphate, then dehydrated in 30%, 50%, 70%, 80%, 90%, 100% acetone for 30 min, respectively. Finally, ultrathin sections were prepared using an ultrathin slicer and stained with uranyl acetate and leaded citrate for examination with a Tecnai Spirit (120 V) transmission electron microscope.

### Determination of chitin and β-1,3-glucan contents in cell walls

Determination methods of chitin were as described previously with modifications [39, 40]. Briefly, conidia (1 × 10^8^) of each strain were grown in 100 mL of synthetic medium for 24 h at 35 °C with shaking at 250 rpm. Removed the culture medium to obtain mycelium, washed with 1 M NaOH for three times, frozen at − 80 °C, vacuum dried and ground into powder for later use. Chitin analysis was performed with three aliquots of 2.5 mg lyophilized mycelia powder as independent samples, and each sample was resuspended in 1.5 mL saturated KOH and incubated for 130 °C for 1 h. After cooled to temperature, 4 mL ice precooling 75% ethanol was added, mixed, and then ice-bathed for 5 min. 150 μL of 13.3% celtite suspension supernatant was added and centrifuged at 6000 rpm for 5 min at 4 °C. The precipitates were washed once with 5 mL pre-cooled 40% ethanol, washed twice with 5 mL pre-cooled double-distilled water, centrifuged at 6000 rpm for 5 min. The precipitates were resuspended with 0.5 mL water and then added with 0.5 mL 5% NaNO_2_ and 0.5 mL 5% KHSO_4_. There were two standard substance: one was 0.2 mL water, one was known as 10 μg/mL hydrochloric acid, and the two standards were added with 0.2 mL 5% NaNO_2_ and 0.5 mL 5% KHSO_4_, mixed once every 5 min for a total of three times, then centrifuged at 6000 rpm for 5 min. Each tube taken supernatant 150 μL, added to the centrifuge tube containing 450 μL H_2_O, added 0.2 mL of 12.5% ammonium sulfamate, mixed for 5 min, then added 0.2 mL of 5 mg/mL of 3-methylbenzthiazolinone-2-hydrazone, reacted at 130 °C for 5 min, after cooled, 0.2 mL of 0.83% FeCl_3_ was added and placed room temperature for 25 min to measured OD 560 nm. Content of β-1,3-glucan between the wild-type and mutant was measured using the aniline blue assay using the previously described [41, 42].

### qRT-PCR analysis

Total RNA was extracted using a column method Total RNA extraction kit (Takara, China) according to the manufacturer’s instructions. Reverse transcription was performed using the HiScript^®^ III RT SuperMix for qPCR (+gDNA wiper) (Vazyme, China) according to the manufacturer’s instructions. All the real-time (qRT -PCR) were performed using a StepOnePlus Real-Time PCR System (Applied Biosystems, USA) and 2 × ChamQ Universal SYBR qPCR Master Mix (Vazyme, China). The reactions and calculations were performed according to standard protocols. Gene transcription levels were determined based on the -ΔΔCT method. The actin gene was used as an endogenous control to normalize the target [43], and three replicates were performed for each sample. All the analyzed genes and primers used for analysis in this work are shown in (Table 2).

**Table 2:** Genes and primers used for qRT-PCR

Gene	Forward primer sequence (5′–3′)	Reverse primer sequence (5′–3′)
*MidA*	GAGTCCGACCAAACAATAAAATCA	CATCGCCATTTGCCGTAAG
*CchA*	AGGTTATCCTCCGCAGTCTCAA	CGATAGCAAACAGAAGCCAGAA
*CrzA*	TGCTCTGGGTCGTCATTTCC	CCCGCTCTTGAGACTCTTCATC
*CnaA*	GTCGGCACTTGACGGACTACTT	GGCAAGGAGCAAAACGATTC
*RodA*	CCGCTGCTGTTCTTGCTTTC	TTGCCATCGTTGCCGTTAC
*PksA*	CGAACGTGAGCTTGAAAGGAT	ACTCGGTGTTTTCACGCTCTTC
*ChsA*	AAGGATGGTTGGAAGAAGATTGTG	CAAAGCCGCCAAAGCATT
*ChsB*	CCGAGGACCGTATCTTGTGTTT	CAGTTTCACCCTTTGAGGCTTT
*ChsC2*	AGTTTATTCTTCAGCGTCGTCGTT	CGCTCCGCCAAATCTGATAG
*ChsD*	ATTCAAGCACGGTGGCAAAC	GTCGGCATAAACCTTGTCAATCA
*ChsC1*	TCGTGGTCTGTTTGGTTTTCG	ATCGTCGCCAACACATCCA
*Actin*	GGTCTGGAGAGCGGTGGTAT	GAAGAAGGAGCAAGAGCAG
*ags1*	ACAACACGAACCGCACCAT	CGGGTGGAACAGGTTTTTGA
*ags2*	TGCTGCGTGCGTTTATGC	TCGTGGATTGGCCGTCTT
*ags3*	TGGGAGGGCACTCACATTC	GCGAGGGACACCGGATTC
*gtb3*	TCCATGGTCGTGATAATGTGAAG	GCCTCCATCCGTTTCATCAT
*agd3*	CTTCACCCACGAGGAGCAA	CCGTTGGCAGTGAAGTGCTT
*ega3*	GGAGCGTCAACGAGCAATG	CCGTGAAGGCAGCGTAGGT
*uge3*	GGACCTTGCGGCTTCTGAT	GGCCAGATTCGTCACATCCT
*uge5*	CCATCCGCGACTACATCCA	CAGGGCCTTCAAGTGACCAT

### Immobilized fermentation and free-cell fermentation

For free-cell fermentation, the flasks were inoculated with 200 μL of spore suspension (10^8^ spores/mL) and then ran at 35 °C with shaking at 300 rpm. After the first batch, 90% of the fermentation broth was removed from the flask and 10% of the fermentation broth was left in the second batch. After adding 100 mL of fresh medium, the second batch was initiated under the same conditions.

For immobilized fermentation experiments, the same conditions for free-cell fermentation were employed except that 1 g/L of the carrier was added into the fermentation medium. At the end of the first batch, the fermented broth was removed from the flask, and then the carrier with the immobilized mycelia was left for the second batch. Subsequent operations were the same as free-cell fermentation. Samples were centrifugated at 8000 rpm, 4 °C for 5 min, and then the supernatants were used for the quantification of citric acid and residual sugar. Citric acid and residual sugar in the supernatant were quantified by high-performance liquid chromatography (Agilent 1100 series; Hewlett-Packard, Palo Alto, CA, USA) with a refractive index detector, using an Aminex HPX- 87H ion exclusion column (300 7.8 mm; Bio-Rad Laboratories, Hercules, CA, USA), with 5.0 mM H_2_SO_4_ used as the mobile phase (0.6 mL/min) at 55 C.

## Supplementary information

**Additional file 1: Figure S1.** Microscopic images of wild-type and mutant conidia adhering to coverslips. The initial conidia concentration was 10^6^/well and samples were observed after 6 h of incubation in 6-well plates.

**Additional file 2: Figure S2**. qRT-PCR result. Heat map of expression levels of genes related to polysaccharide synthesis in biofilms of WT, ∆*MidA*, ∆*CchA*, ∆*CrzA* and ∆*CnaA* strains.

**Additional file 3: Table S1.** Sequence of the oligonucleotide primers used for gene knockout in this study.

**Additional file 4: Figure S3.** qRT-PCR verification of the mutant strains and complemented strains. Values and error bars represent the mean and the s.d. (*n *= 3).

**Additional file 5: Table S2.** Sequence of the oligonucleotide primers used by plasmids was constructed in this study.

## Data Availability

The authors promise the availability of supporting data.

## Abbreviations

CSP: Calcineurin signaling pathway
Hyg: Hygromycin
GAG: Galactosaminogalactan
CV: Crystal violet
SEM: Scanning electron microscopy
CR: Congo red
CFW: Calcofluor white
TEM: Transmission electron microscope
