# Supplementary figures and images for: Calcineurin signaling pathway influences Aspergillus niger biofilm formation by affecting hydrophobicity and cell wall integrity
Source: Biotechnol Biofuels. 2020 Mar 16;13:54. doi: 10.1186/s13068-020-01692-1 (PMC7075038; doi:10.1186/s13068-020-01692-1)

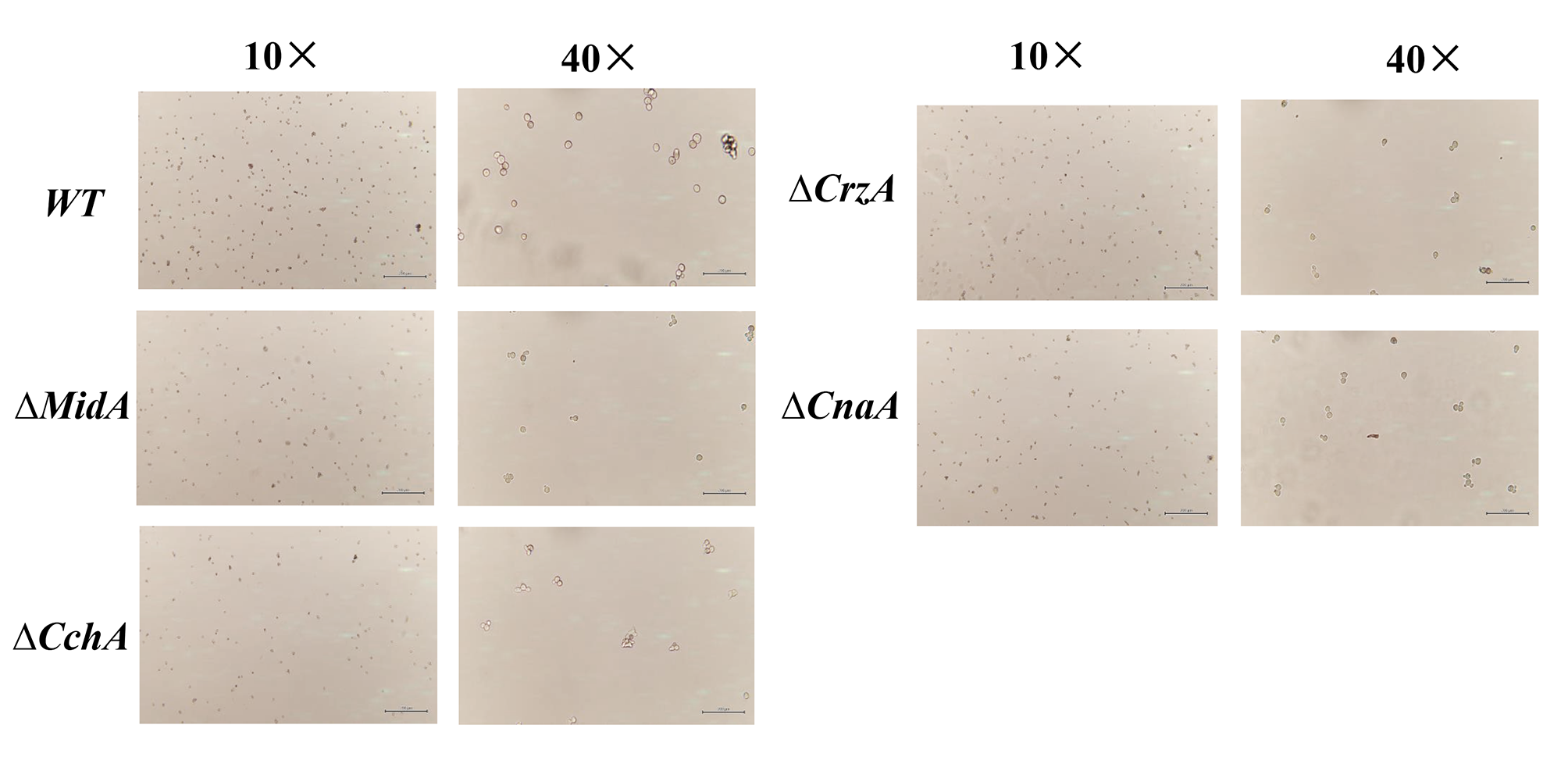

Supplement: Supplementary file 1 — Additional file 1: Figure S1. Microscopic images of wild-type and mutant conidia adhering to coverslips. The initial conidia concentration was 106/well and samples were observed after 6 h of incubation in 6-well plates. [file 13068_2020_1692_MOESM1_ESM.tif]

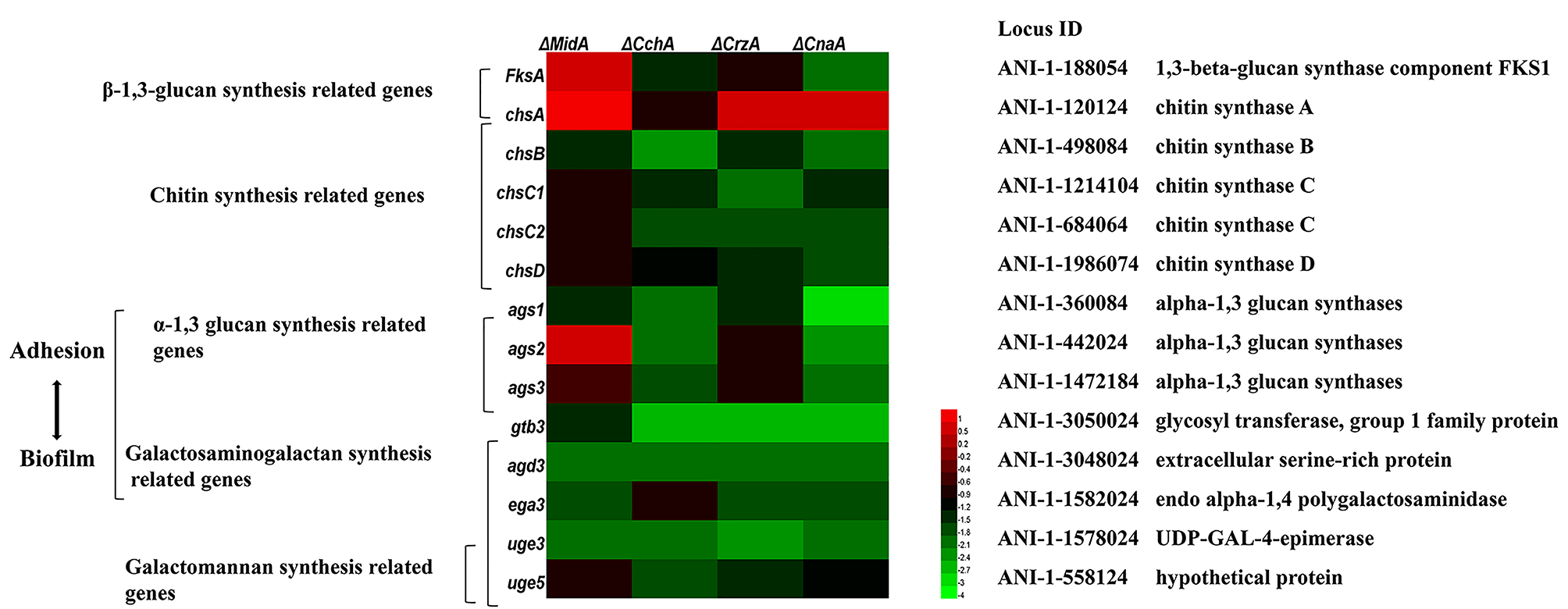

Supplement: Supplementary file 2 — Additional file 2: Figure S2. qRT-PCR result. Heat map of expression levels of genes related to polysaccharide synthesis in biofilms of WT, ∆MidA, ∆CchA, ∆CrzA and ∆CnaA strains. [file 13068_2020_1692_MOESM2_ESM.tif]

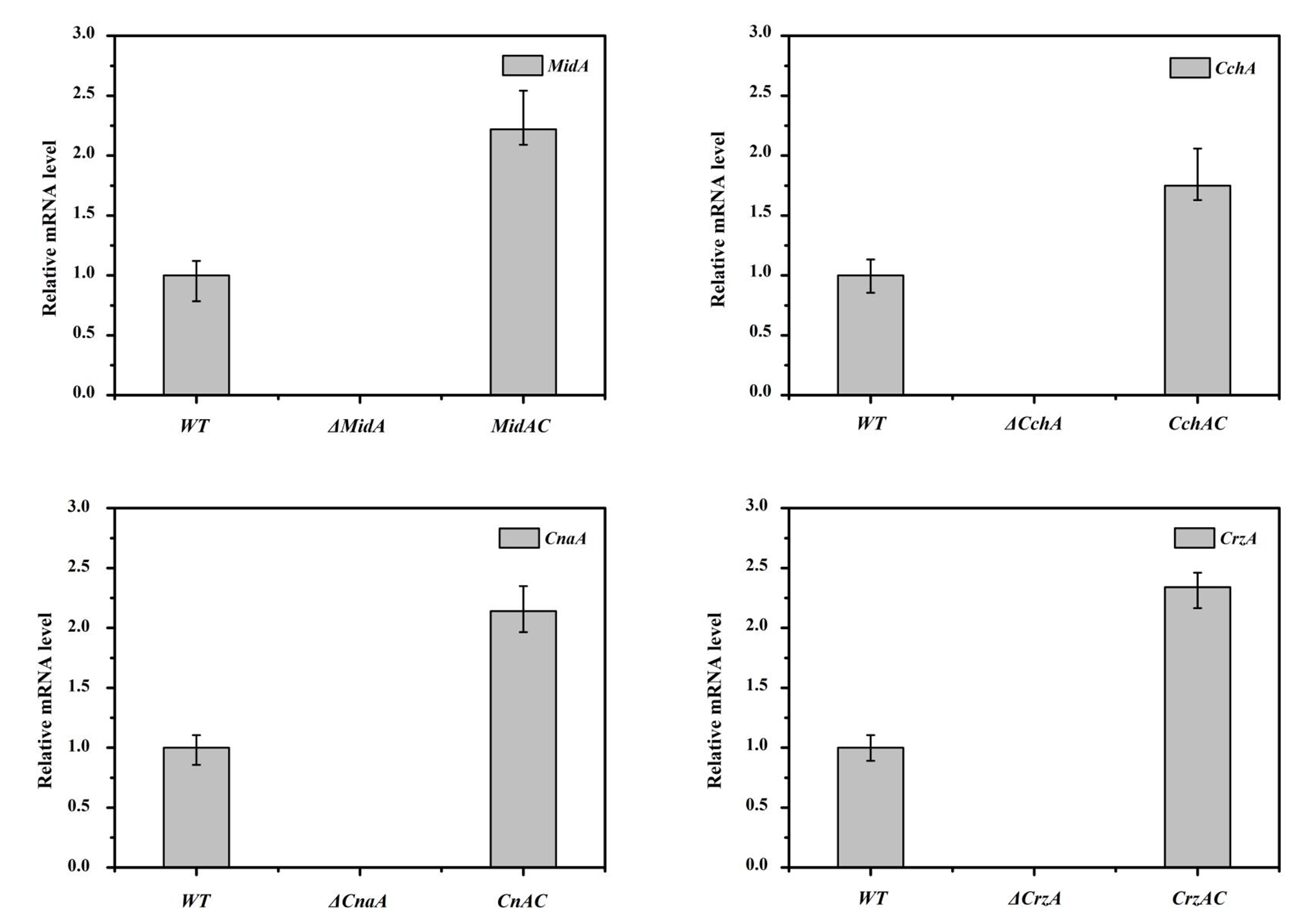

Supplement: Supplementary file 4 — Additional file 4: Figure S3. qRT-PCR verification of the mutant strains and complemented strains. Values and error bars represent the mean and the s.d. (n = 3). [file 13068_2020_1692_MOESM4_ESM.tif]
