# Supplementary material for: Calcineurin signaling pathway influences Aspergillus niger biofilm formation by affecting hydrophobicity and cell wall integrity
Source: Biotechnol Biofuels. 2020 Mar 16;13:54. doi: 10.1186/s13068-020-01692-1 (PMC7075038; doi:10.1186/s13068-020-01692-1)
Supplement: Supplementary file 3 — Additional file 3: Table S1. Sequence of the oligonucleotide primers used for gene knockout in this study. [file 13068_2020_1692_MOESM3_ESM.docx]

**Additional file 3: Table** **S1** Sequence of the oligonucleotide primers used for gene knockout in this study

| Primer name | Primer sequence | source |
| --- | --- | --- |
| *MidA*-up-F | CCAACCGATGATGGCTGTCT | This work |
| *MidA*-up-R | GGTGGAGGCGGCGGATTTTAGG  TGCCGGAACGTAACAACT | This work |
| *MidA*-down-F | CCCACTCCACATCTCCACTCGAC  GGAATCATGGCTCGTGAGG | This work |
| *MidA*-down-R | GATATTCGCCTGCTTGGCGG | This work |
| Hyg-*MidA*-F | AGTTGTTACGTTCCGGCACCTAA  AATCCGCCGCCTCCACC | This work |
| Hyg-*MidA*-R | CCTCACGAGCCATGATTCCGTCG  AGTGGAGATGTGGAGTGGG | This work |
| *MidA*-F | GCCTGGCAGGATTCCAGTGA | This work |
| *MidA*-R | TGGTAGATCGCGTAGCGAGG | This work |
| *CchA*-up-F | CGGCGATGACGACGAGACTA | This work |
| *CchA*-up-R | GGTGGAGGCGGCGGATTTTAA  GTCGTCAGAGTGCTAACCG | This work |
| *CchA*-down-F | CCCACTCCACATCTCCACTCGAC  TACACGCTCGAGATTATCGCC | This work |
| *CchA*-down-R | GCGAGGCCATGCTAACTTGT | This work |
| Hyg-*CchA*-F | CGGTTAGCACTCTGACGACTTAA  AATCCGCCGCCTCCACC | This work |
| Hyg-*CchA*-R | GGCGATAATCTCGAGCGTGTAG  TCGAGTGGAGATGTGGAGTGGG | This work |
| *CchA*-F | CGAACCACAACCACGGAGGA | This work |
| *CchA*-R | TTCATCCTCCGAGACATCAAAGCT | This work |
| *CnaA*-up-F | ACCGGAGTGGAAGGAGCTTG | This work |
| *CnaA* -up-R | GGTGGAGGCGGCGGATTTTAG  GTTGCGAGGAGGCGAAGAA | This work |
| *CnaA* -down-F | CCCACTCCACATCTCCACTCGAT  CATCCACAACAGCGTCCGC | This work |
| *CnaA* -down-R | CCATACGCATCCAATGCATCC | This work |
| Hyg-*CnaA*-F | TTCTTCGCCTCCTCGCAACCTAA  AATCCGCCGCCTCCACC | This work |
| Hyg- *CnaA* -R | GCGGACGCTGTTGTGGATGATC  GAGTGGAGATGTGGAGTGGG | This work |
| *CnaA* -F | GAGGAAGTGTGGTGGTGGTGAG | This work |
| *CnaA* -R | CAACCGGCTTCTCACTCTCCAA | This work |
| *CnaA* -up-F | TGGATCCTGCAGTCTGCCTT | This work |
| *CrzA*-up-R | GGTGGAGGCGGCGGATTTTAA  GATACCACGCGATGCAAGG | This work |
| *CrzA* -down-F | CCCACTCCACATCTCCACTCGAC  CGGCGAAGAATCTCAACCT | This work |
| *CrzA* -down-R | CAATGGGCATAGGACTGAACCT | This work |
| Hyg- *CrzA* -F | CCTTGCATCGCGTGGTATCTTAA  AATCCGCCGCCTCCACC | This work |
| Hyg- *CrzA* -R | AGGTTGAGATTCTTCGCCGGTC  GAGTGGAGATGTGGAGTGGG | This work |
| *CrzA* -F | TCTAGGCAGCGTTCGCAAGG | This work |
| *CrzA* -R | GCAGAAAGGTCTACCGCACC | This work |
| Hyg-F | TTCGACAGCGTCTCCGACCT | This work |
| Hyg-R | ACACAGCCATCGGTCCAGAC | This work |
