# Supplementary material for: Calcineurin signaling pathway influences Aspergillus niger biofilm formation by affecting hydrophobicity and cell wall integrity
Source: Biotechnol Biofuels. 2020 Mar 16;13:54. doi: 10.1186/s13068-020-01692-1 (PMC7075038; doi:10.1186/s13068-020-01692-1)
Supplement: Supplementary file 5 — Additional file 5: Table S2. Sequence of the oligonucleotide primers used by plasmids was constructed in this study. [file 13068_2020_1692_MOESM5_ESM.docx]

**Additional file 5: Table S2** Sequence of the oligonucleotide primers used by plasmids was constructed in this study

| Gpda-F | GAGGTAATCCTTCTTTCTAGATAAAATCCGC  CGCCTCCACC | This work |
| --- | --- | --- |
| Gpda-*MidA*-R  *MidA*-G418-F | GGAGATGGGCCTGAGGGGGAAAAGAAAGAG  AAAAGAAAAGAGCAG  CTGCTCTTTTCTTTTCTCTTTCTTTTCCCCCTC  AGGCCCATCTCC | This work  This work |
| *MidA*-G418-R | CAGTACACGAGGACTTCTAGAAAAGAAAAG  ACGAATATTTTAGAGGAT | This work |
| Gpda-*CchA*-R  *CchA*-G418-F  *CchA*-G418-R  Gpda-*CrzA*-R | GGTCATGGCTACTTGAAGCCATGGGAAAAG  AAAGAGAAAAGAAAAGAGCAG  CTGCTCTTTTCTTTTCTCTTTCTTTTCCCATG  GCTTCAAGTAGCCATGACC  CAGTACACGAGGACTTCTAGATCACTCCCC  CGATCGCC  ACAGCAATGGAGAATTCGCGGGGAAAAGA  AAGAGAAAAGAAAAGAGCAG | This work  This work  This work  This work |
| *CrzA*-G418-F | CTGCTCTTTTCTTTTCTCTTTCTTTTCCCCGC  GAATTCTCCATTGCTGT | This work |
| *CrzA*-G418-R | CAGTACACGAGGACTTCTAGATCATGCTCT  CCAATCCGTTTCT | This work |
| Gpda-*CnaA*-R | GGAGCGAAATAGGGTTGGAAAAATTGGGG  AAAAGAAAGAGAAAAGAAAAGAGCAG | This work |
| *CnaA*-G418-F | CTGCTCTTTTCTTTTCTCTTTCTTTTCCCCAA  TTTTTCCAACCCTATTTCGCTCC | This work |
| *CnaA*-G418-R | CAGTACACGAGGACTTCTAGATTATGCAAT  ACAGTAATCTCTCGCGAC | This work |
